# PM-profiler: a high-resolution and fast tool for taxonomy annotation of amplicon-based microbiome

**DOI:** 10.1128/spectrum.00695-24

**Published:** 2024-06-24

**Authors:** Haobo Shi, Guosen Hou, Sikai Jiang, Xiaoquan Su

**Affiliations:** 1 College of Computer Science and Technology, Qingdao University, Qingdao, Shandong, China; Chengdu University, Chengdu, China

**Keywords:** microbiome, bioinformatics, profiling, amplicon, algorithm, software

## Abstract

Amplicon sequencing stands as a cornerstone in microbiome profiling, yet concerns persist regarding its resolution and accuracy. The enhancement of reference databases and annotations marks a new era for 16S rRNA-based profiling. Capitalizing on this potential, we introduce PM-profiler, a novel tool for profiling amplicon short reads. PM-profiler is implemented by C++-based advanced algorithms, such as pre-allocated hash for reference construction, hybrid and dynamic short-read matching, big-data-guided dual-mode hierarchical taxonomy annotation strategy, and full-procedure parallel computing. This tool delivers species-level resolution and ultrafast speed for large-scale microbiomes, surpassing alignment-based approaches and the Naïve-Bayesian model. Furthermore, recognizing the global uneven distribution of microbes, we delineate optimal annotation strategies for each sampling habitat based on microbial patterns over 270,000 microbiomes. Integrated with the established workflow of Parallel-Meta Suite and the latest curated reference databases, this endeavor offers a swift and dependable solution for high-precision microbiome surveys.

**IMPORTANCE:**

Our study introduces PM-profiler, a new tool that deciphers the complexity of microbial communities. With advanced algorithms, flexible annotation strategies, and well-organized big-data, PM-profiler provides a faster and more accurate way to study on microbiomes, paving the way for discoveries that could improve our understanding of microbiomes and their impact on the world.

## INTRODUCTION

Microbiome analysis has revolutionized our understanding of microbial communities inhabiting diverse environments, ranging from different sites in the human body and animals to ecosystems (1–5). Due to the vast volume, diverse environmental sources of existing data, and low cost, amplicon sequencing, primarily targeting the 16S rRNA gene, has emerged as a pivotal technique in unraveling the taxonomic composition and functional potential of microbiomes (6, 7). However, despite its widespread adoption, concerns regarding the resolution and accuracy of amplicon sequencing persist within the scientific community (8).

In recent years, the demand for high-resolution profiling of microbiomes, particularly at the species level, has surged (9, 10). The gradual refinement of reference databases (11, 12) has laid the groundwork for more precise characterization of microbial communities across various niches. However, as the field advances, researchers seek methods that not only provide accurate taxonomic assignments but also offer rapid processing of large-scale data sets. While widely used sequence matching-based approaches like Vsearch (13) and BLAST (14) rely on best matches, which may introduce false positives, model-based classifiers like Naïve-Bayesian (15) face challenges such as insufficient annotation depth, sensitivity, and computing throughput.

Acknowledging the evolving landscape of microbiome analysis, we introduce PM-profiler, a state-of-the-art tool designed to overcome the inherent limitations of current amplicon sequencing methodologies. PM-profiler harnesses a suite of sophisticated algorithms and endeavors to provide unparalleled resolution and processing speed for microbiome data sets of varying complexities. Furthermore, by synthesizing insights gleaned from extensive microbiome big-data (16), we aim to delineate optimal annotation strategies tailored to diverse environmental contexts. Through seamless integration with established workflows such as Parallel-Meta Suite (17) and curated reference databases like Greengenes2 and RefSeq, PM-profiler represents a significant advancement toward achieving rapid and reliable microbiome data mining across a spectrum of research domains.

## MATERIALS AND METHODS

### The PM-profiler framework

**Fig 1:**
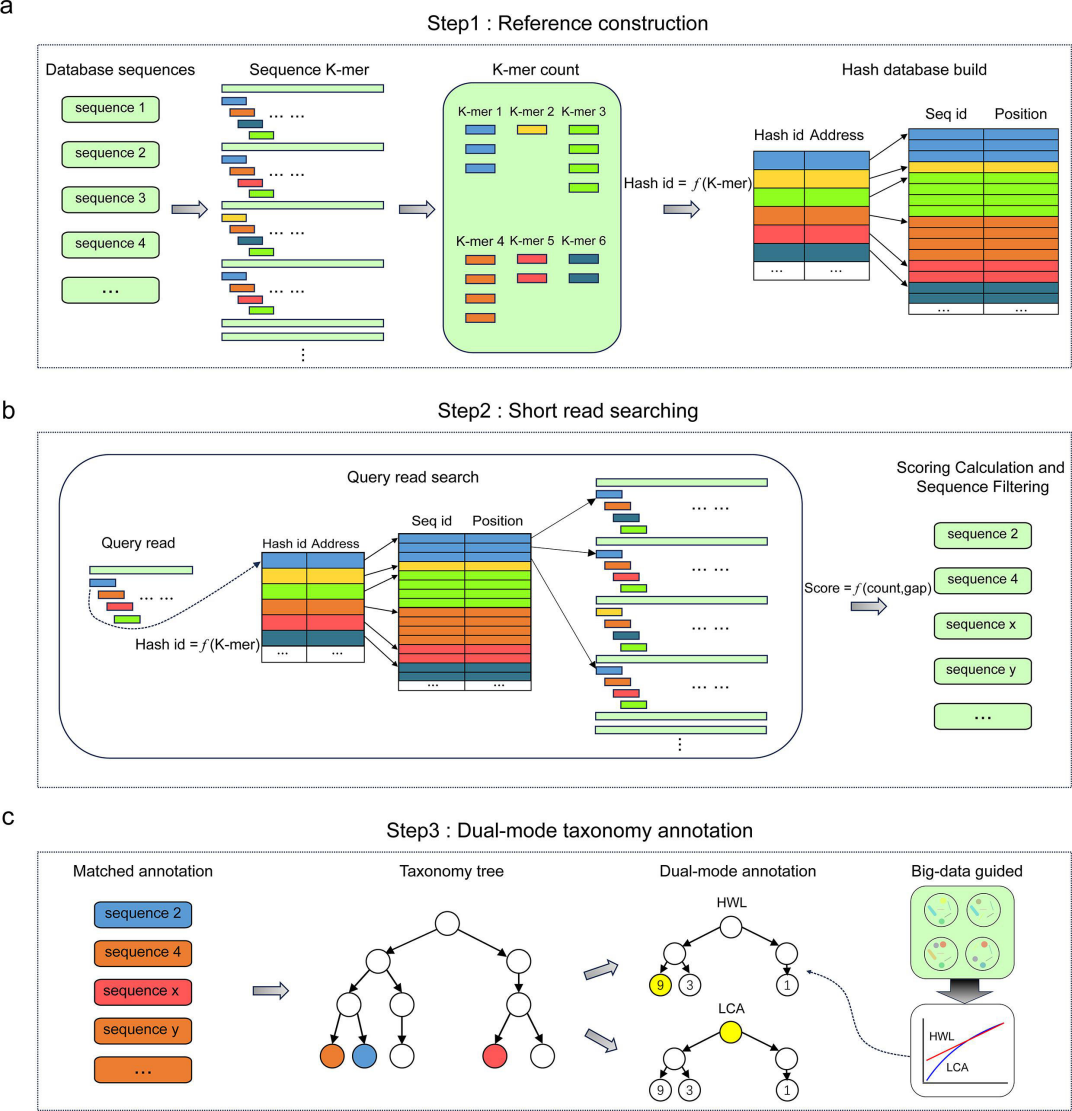
Overall schema of PM-profiler. (**A**) Reference construction using pre-allocated space k-mer hash. (**B**) Rapid short-read searching. (**C**) Dual-mode hierarchical taxonomy annotation strategy.

The primary goal of PM-profiler is to accurately and efficiently parse taxonomy annotations for amplicon short reads against a reference database with high resolution. It operates through three pivotal steps (Fig. 1). Firstly, in the database load phase, it constructs a pre-allocated space k-mer hash for all reference sequences within a given database (Fig. 1A). Subsequently, when presented with a query sample, PM-profiler systematically searches each short read utilizing hybrid scoring metrics to identify multiple matches (Fig. 1B). These matches serve as the basis for detailed taxonomy annotation, which is parsed using dual-mode hierarchical strategies with big-data-guided recommendation (Fig. 1C). PM-profiler is coded in C++, with OpenMP-based parallelization across all three steps (refer to Supplementary Materials for details). Its application is versatile: it can function independently as a standalone profiler or be seamlessly invoked within the workflow of the Parallel-Meta Suite (PMS) for comprehensive microbiome data mining.

### Reference construction using pre-allocated space k-mer hash

Initially, PM-profiler extracts all k-mers (with a default k value of 15 that balanced specificity and memory usage, refer to Supplementary Materials for details; Fig. S1) from database sequences (e.g., full-length 16S rRNA gene) and stores in a contiguous-space hash table. Specifically, hash index keys are calculated using a k-digit quaternary transformer hash function (*equation 1*) for each k-mer *S*, in which *S_i_* represents the *i*th nucleotide in this k-mer.


(1) $$Hash\left(S\right)=\;\sum_{i=1}^{k}f\left(S_{i}\right)*4^{k-i},\;here\;f\left(S_{i}\right)\;=\left\{ \begin{array}{c} \;\;0\;,\;\;\;\;if\;S_{i}\;={\;}^{\prime }\;A^{\prime }\;\;\;1\;,\;\;\;\;if\;S_{i}\;={\;}^{\prime }\;C^{\prime }\\ \;\;2\;,\;\;\;\;if\;S_{i}\;={\;}^{\prime }\;G^{\prime }\;\;\;3\;,\;\;\;\;if\;S_{i}\;={\;}^{\prime }\;T^{\prime } \end{array}\right.$$

The hash values retain the source sequences and their relative positions of an index (i.e., a k-mer; Fig. 1A). Since a k-mer can stem from multiple sequences and the number of source sequences differ among distinct k-mers, PM-profiler systematically scans the database to ascertain the distribution of k-mers and pre-allocates space accordingly (refer to Supplementary Materials for details). This approach minimizes storage requirements and enhances the efficiency of retrieving hash values.

### Rapid short-read searching with dynamic matching

Upon encountering each query short-sequence read, PM-profiler decomposes it into k-mers and transforms them into hash indices following the same procedure as with the reference sequences. These hash indices are subsequently mapped in the hash table to retrieve the candidate matched reference sequences (Fig. 1B). Here, we set a matching score metrics, which evaluates the matching degree between the query read and the reference by considering both their maximum number of common k-mers and the minimum of relative position difference. Notably, the matching score is not calculated in an end-to-end way but dynamically maintained to drop candidates with low matching probability. This hash-based approach avoids the complexity of the traditional sequence alignment procedure, while also mitigates the effects of mismatches, insertions, and deletions (refer to Supplementary Materials for details). Consequently, this step efficiently selects highly matched references of each short read in the query microbiome sample for taxonomy annotation.

### Dual-mode hierarchical taxonomy annotation strategy

After identifying matched reference sequences for a short read, PM-profiler extracts their tiered annotations to construct a hierarchical taxonomy tree. The number of sequences associated with each node is considered as the weight, forming the basis for annotation (Fig. 1C). The final annotation of a short read is determined using two alternative strategies: HWL (Highest Weighted Leaf): the leaf node with the highest weight is identified, ensuring species-level taxonomy resolution and sensitivity; and LCA (Lowest Common Ancestor): all leaf nodes are traced to their lowest common ancestor to ensure purity and reliability of the annotation. Here, we provide two principles to select the annotation strategy (Table 1):

*a. Requirement-based*: the HWL method offers a balanced overall F1 score while focusing on completeness in most cases. In PM-profiler, we set it as the default strategy for large-scale microbial surveys and data mining. The LCA method, being conservative in pursuing recall and purity, is more reliable in recognizing specific microbes such as pathogens or biomarkers.

*b. Big-data guided and habitat-oriented*: by assessing the consistency of the same species across different amplicon variation regions and analyzing abundant species across 275,793 microbiomes from MSE (Microbiome Search Engine; Table S1), we summarized the optimal annotation strategy for 17 typical habitats (Table 1; Fig. S2, and Table S2). In essence, for a given habitat, HWL is recommended if its abundant species exhibit high consistency in 16S rRNA for enhanced annotation resolution; otherwise, we suggest LCA to ensure purity.

**TABLE 1:** Characters of two annotation strategies of PM-profiler

Annotation strategy	HWL	LCA
Advantage	Higher overall F1 score on species level; higher taxonomy resolution; assigned with a reference database id	Higher purity and reliability
Example requirement	The default strategy; microbial pattern survey on large scale; close reference-based downstream analysis	Verification of specific species, e.g., pathogen or markers
Suggested habitat (V4 variable region)	Human (gut, skin, nose, oral, and urogenital), building, food, freshwater, lake, marine, mammal animal, milk, non-mammal animal, river, etc.	Plant, soil, sand, etc.
Suggested habitat (V3–V5 variable region)	Human (gut, skin, nose, oral, and urogenital), food, mammal animal, milk, non-mammal animal, etc.	Building, freshwater, plant, soil, sand, river, marine, lake, etc.

## RESULTS AND DISCUSSION

### Evaluation of annotation by simulated data set

In this study, we generated an artificially simulated data set to assess the performance of PM-profiler and compared it with other profiling tools, including sequence matching approaches such as Vsearch (13) and BLASTn (14), as well as model-based classifiers like Naïve-Bayesian. Performance was evaluated based on completeness, purity, and F1 score metrics. The simulated data set mirrored the species distribution of six distinct habitats (Table S3; refer to Supplementary Materials for details), each comprising five samples, totaling 30 samples. These habitats encompassed human gut, human skin, human oral, mouse gut, marine, and soil environments. Using 515F and 806R primers, we extracted 300-bp pair-end fragments from the V4 region of RefSeq whole genomes. Additionally, we introduced sequencing errors typical of Illumina platforms. Short reads underwent species-level taxonomy annotation through various approaches using the RefSeq 16S amplicon database (10, 11), which was independent of the RefSeq genomes utilized for short-read simulation.

**Fig 2:**
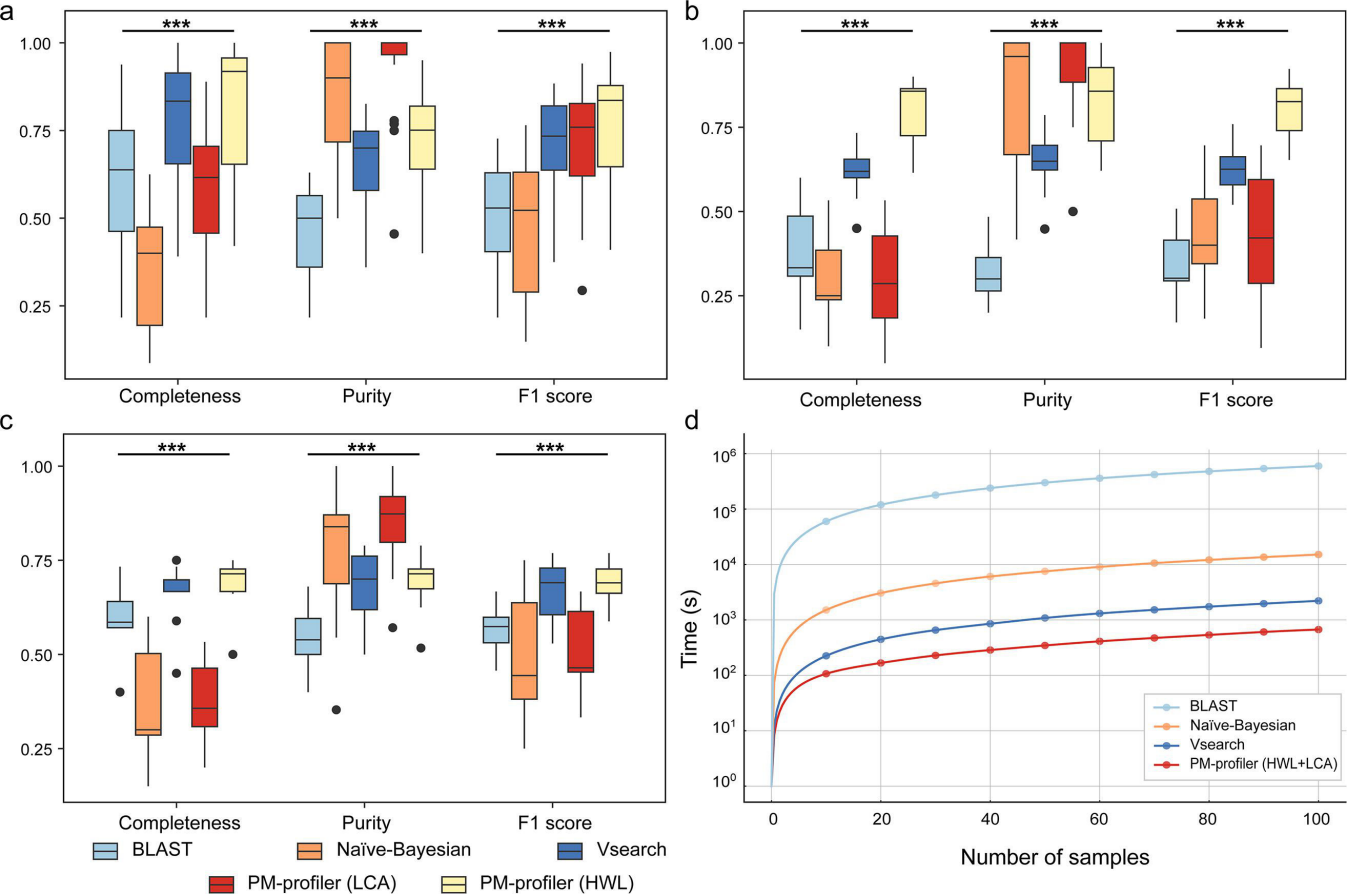
Performance evaluation and comparison of different profilers. (**A**) Species-level taxonomy parsed from RefSeq database on simulated data sets. (**B**) Species-level taxonomy parsed from the RefSeq database on mock data sets. (**C**) Species-level taxonomy parsed from the Greengenes2 database on mock data sets. (**D**) Running time using different number of samples. *** denotes *P* value < 0.01 by two-tailed rank sum test.

Results depicted in Fig. 2A indicate that PM-profiler in HWL mode achieved the highest overall F1 score and completeness among all methods, while LCA mode maintained the highest purity. Notably, the performance of the two annotation modes varied across different habitats (Fig. S3). For instance, HWL mode significantly outperformed other methods in human gut and marine environments, whereas the LCA method yielded higher F1 scores in soil samples. These results were highly consistent with our recommendation based on big data in Table 1. Such discrepancy can be attributed to the sequence redundancy and consistency of species within the reference database. For example, microbes prevalent in human gut, marine, and mouse gut environments exhibit stronger consistency within the same species on the amplified variation region, resulting in superior performance under HWL mode; conversely, the diverse 16S rRNA gene sequences among abundant species in soil render the LCA method more suitable for these environments.

### Evaluation of annotation by mock microbiomes

In addition to simulated data, we compared different approaches using a mock data set comprising 10 microbiomes (Table S4) sequenced from artificially mixed real bacterial cultures provided by Mockrobiota (18). Short reads were annotated on the species level using both the Greengenes2 and RefSeq amplicon databases. Figure 2B and C illustrated the species-level annotation results of various approaches using the two databases, respectively, showing a highly consistent trend. The HWL method exhibited the highest overall F1 score among all approaches on this data set, making it a default strategy for most scenarios. Meanwhile, the LCA method demonstrated exceptional purity, which is crucial for identifying specific microbes.

### Evaluation of running speed

The speed of annotation holds critical significance in large-scale microbiome studies. Here, we benchmarked the efficiency of different tools in profiling using a simulated data set with varying numbers of microbiomes. Each microbiome contained 10,000 V4 region amplicon reads randomly selected from the Greengenes2 database. We configured PM-profiler to output results for both annotation strategies. All tests were conducted using an exclusive server with a 64-core CPU. The resulting time depicted in Fig. 2D showcased PM-profiler’s superior speed compared with all other tools, especially with over 50 samples. This demonstration of PM-profiler’s efficiency is pivotal, highlighting its potential to expedite data processing in high-throughput metagenomic studies and facilitating quicker, more informed research outcomes.

### Conclusion

In this study, we introduced PM-profiler, a novel tool designed to address the challenges associated with amplicon sequencing-based microbiome profiling. PM-profiler represents a significant advancement in microbiome analysis, offering researchers a reliable, efficient, and flexible tool for high-resolution taxonomy annotation. Its integration with established workflows and reference databases further enhances its utility in diverse research applications. We anticipate that PM-profiler will catalyze advancements in microbiome research and contribute to a deeper understanding of microbial communities in various ecosystems and biological contexts.

## Data Availability

The source code of PM-profiler software is available at https://github.com/qdu-bioinfo/PM-profiler, and the latest Parallel-Meta Suite workflow integrated with PM-profiler is available at https://github.com/qdu-bioinfo/parallel-meta-suite. Simulated samples are available at the NCBI SRA database under BioProject accession number PRJNA1087515.

## SUPPLEMENTAL MATERIAL

Supplemental figures spectrum.00695-24-s0001.docx Fig. S1 to Fig. S3; supplemental methods.

Supplemental material spectrum.00695-24-s0002.xlsx Rawdata of figures.

Supplemental tables spectrum.00695-24-s0003.xlsx Tables S1 to S4.

ASM does not own the copyrights to Supplemental Material that may be linked to, or accessed through, an article. The authors have granted ASM a non-exclusive, world-wide license to publish the Supplemental Material files. Please contact the corresponding author directly for reuse.
