# Supplementary material for: PM-profiler: a high-resolution and fast tool for taxonomy annotation of amplicon-based microbiome
Source: Microbiol Spectr. 2024 Jun 24;12(8):e00695-24. doi: 10.1128/spectrum.00695-24 (PMC11302061; doi:10.1128/spectrum.00695-24)
Supplement: Supplemental figures — Fig. S1 to Fig. S3; supplemental methods. [file spectrum.00695-24-s0001.docx]

**Supplementary Materials for “PM-profiler: a high-resolution and fast tool for taxonomy annotation of amplicon-based microbiome”**

**Supplementary Methods**

**Reference construction**

The database of PM-profiler is an inverted index hash table, specifically comprising two linear hash tables: an actual data table, $H(D)$, wherein the sequence storage order is determined by the sorted hash values of k-mers, and an index table, $H(K)$, employed to store the starting addresses of the data corresponding to each k-mer within $H(D)$ (**Fig. 1a**). During the search process, by calculating the hash value of a k-mer, one can swiftly locate the address of the data corresponding to that k-mer in $H(K)$and sequentially traverse $H(D)$ to identify all sequences containing this k-mer and the positions of the k-mer therein.

Once a database is specified by the user, PM-profiler segments the input sequence into k-mers and tallies their occurrences. Each k-mer is then converted into a hash value $K$ using a quad-hash function. These values are stored as keys in a hash table $H(K)$, with the k-mer count as the associated value, to facilitate database space pre-allocation. Upon completing the counting process, PM-profiler accumulates the k-mer occurrences to update the values in $H(K)$ for subsequent addressing. Subsequently, PM-profiler reload the database and constructs an additional hash table $H(D)$,which serves as the structural backbone for the final database storage. For every input sequence S, PM-profiler breaks it down into k-mers and calculates the corresponding hash value $K$, then searches for matching k-mers within $H(K)$. Upon successful matching, the hash value $K$'s corresponding value is used as a designated address in $H(D)$ to store the sequence name S and k-mer position while updating the value associated with $K$.

**K-mer size selection**

Longer k-mer size provides higher specificity but also requires more memory consumption, vice versa. Specifically, the specificity can be evaluated by the number of sequences with duplicated k-mers in the reference, while the memory consumption can also be estimated by the hash size. Therefore, by comparing the performance of different k-mers from 8 to 24 in RefSeq database (**Fig. S1**), we selected 15 as the default k-mer size, which achieves a balance between the specificity and memory usage on regular PCs.

**Short read mapping algorithm**

For each query sequence denoted as $Q$, PM-profiler initiates by constructing a scoring hash table $H(R)$, indexed by the reference sequence $R$. This table scores based on the count of overlapping k-mers $(R_{count})$, the position of the current k-mer $(R_{pos})$, and the gap $(R_{gap})$ between successive k-mer positions within $R$. PM-profiler then partitions $Q$ into k-mers and calculates their respective hash values utilizing a quad-hash function. Sequentially, PM-profiler accesses each k-mer's hash value $K$, conducting a two-fold retrieval from $H(K)$ and $H(D)$ to pinpoint all corresponding sequences in the database, including their loci within $H(D)$. For every sequence $R$, within the database, the auxiliary table $H(R)$, is updated to reflect the new overlap count$R_{count}$, the current k-mer's position $R_{pos}$, and to compute the interval $R_{gap}$ relative to the preceding k-mer's location. Upon the completion of the search phase, PM-profiler employs a similarity formula to compute the similarity across all sequences, thereby filtering and outputting the results for subsequent annotation. Specifically, given the size of the k-mer $k$, the length of the query sequence $L_{Q}$, the total number of k-mer's $Q_{count}$, the number of k-mers matching the reference sequence $R_{count}$, and the total gap sum $R_{gap}$, similarity is determined by *equation S1*:

| $R_{Similarity}=1-\frac{\left\lfloor\frac{Q_{count}-R_{count}}{k} \right\rfloor+R_{gap}\mathrm{mod} k}{L_{Q}}$ | *(S1)* |
| --- | --- |

PM-profiler calculates the maximum similarity among all reference sequences and compares it with a predetermined similarity threshold. Should the resultant similarity exceed this threshold, sequences exhibiting this maximal similarity metric are designated as inputs for the subsequent steps (**Fig. 1b**).

**Taxonomic annotation strategy**

Within the collection of sequences derived from search outcomes, PM-profiler seeks their annotations within an annotation file to construct a pruned taxonomy subtree, wherein the weight of each node is determined by the ratio of the number of sequences possessing that annotation to the total number of sequences. This serves as the foundation for annotation statistics. Sequences that cannot be classified, for instance, those with species-level annotations designated as *s__*, are excluded from the tree. Upon the tree's completion, two distinct strategies are employed for re-annotation based on the taxonomy tree: The Highest Weight Label (HWL) method initiates from the leaf nodes and progresses upward layer by layer, identifying and outputting the node with the highest weight above a predetermined threshold at each level. Conversely, the Lowest Common Ancestor (LCA) method commences at the root node and traverses to the downward layers until a level is reached where more than two leaf nodes exist, thereby ensuring the consistency of the output across all sequences (**Fig. 1c**).

**Simulation of microbiome dataset**

We established six groups of simulated V4 region datasets derived from diverse habitats including human gut, human skin, human oral cavity, mouse gut, marine environments, and soil. Utilizing a priori annotation information from WGS sequences obtained from the MSE and SRA databases, common species compositions corresponding to each environment were selected from the data labeled with habitats to compose the species pool for each environment (**Table S1**). For every habitat, we simulated five amplicon datasets, with the original species randomly selected from the species pool, to evaluate the annotation effectiveness for each species and to ensure reliability. Specifically, in the creation of each simulated dataset, each species had a 50% probability of being chosen as the original species for that dataset. Upon selection, no more than five genomes for the species were randomly extracted from the full genomes in the ref-seq database to serve as the original sequences for the simulated data, The V4 region of the dual-end amplicons was then extracted using the primers 515F (GTGYCAGCMGCCGCGGTAA) [1] and 806R (GGACTACNVGGGTWTCTAAT) [2]. Upon extraction, sequencing errors were introduced into the simulated amplicon sequences by analyzing the error model data from Illumina sequencers. The quality $Q\left( n \right)$of the $n-th$ base in short-read sequences was generated according to *equation S2* representing the sequencing error profile.

| $Q\left( n \right)=\left\{ \begin{aligned} 37-1.88\times{10}^{-4}\times n^{2}, 5^{'}\sim3' \\ 33-1.88\times{10}^{-4}\times n^{2}, 3^{'}\sim5' \end{aligned} \right.$ | *(S2)* |
| --- | --- |

For each base, a random floating-point number in the range of (0,1) is generated. If this number is less than *10^-(Q(n)/10)^* then the base is randomly replaced by an insertion, deletion, or mutation with respective probabilities of 5%, 5%, and 90%. The average error rate for the 5' to 3' direction of paired-end sequencing is less than 0.5%, while for the 3' to 5' direction, it is less than 1.0%. Each 16S sequence underwent 100 bootstrap iterations to ensure sequencing errors were reflected in the sequence. Additionally, sequencing quality typically deteriorates at the beginning and end of sequencing due to voltage instability, affecting 3 to 10 bp, necessitating correction or removal. Considering the presence of adapter and primer sequences, the sequencing read length is generally less than the actual sequencing read length of the sequencing reagents. Consequently, this study removed a total of 30 bp from each sequencing sequence, comprising approximately 20 bp of primer sequences and 10 bp of terminal sequences.

**Annotation result analysis**

For mock data (**Table S2**), to emulate the actual sequencing process, we utilized Cutadapt [3] to remove primer sequences from the samples, followed by employing DADA2 [4] for sequence filtering. The trimming thresholds for both ends were set to 5 to eliminate low-quality bases, and the resultant sequences served as inputs for annotation by various tools. In the case of simulated data, without the impact of unknown variables inherent to real sequencing, and to test theoretical errors caused by sequencing inaccuracies and database redundancies, we directly executed paired-end merging using FLASH [5] with parameters set to a minimum overlap of 10, maximum overlap of 300, and maximum error rate of 0.15. Subsequently, the merged sequences were directly annotated using various tools.

Annotations were performed using the following tools and settings:

1. BLASTn, version 2.10.1+. It was run with maxaccept set to 1, e-value at 1e-8, and wordlength at 15.
2. Vsearch, version v2.7.0. Annotation was conducted in usearch-global mode with wordlength set to 15, similarity at 0.99, and sequence coverage at 0.99.
3. Naïve-bayes classifier. Qiime feature-classifier classify-sklearn, version 2020.11, employing default parameters for annotation.
4. PM-profiler, version v1.0. The output mode was set to HWL+LCA for annotation.

For sequence alignment results from BLAST and Vsearch, the annotations were derived from corresponding sequences in the annotation file. NCBI-taxid served as the standard for correct annotation. Sequences with ambiguous species-level annotations, unable to obtain an NCBI-taxid (e.g., *s__*), were treated as unclassified species within the corresponding genus to ascertain the NCBI-taxid. For the mock data, the annotation results were filtered at a rate of 0.01% (mock12 was not filtered, as it contains species with abundances less than 0.01%). For simulated data, the results were filtered at a percentage of 0.1%. The annotated result's taxid was input into CAMI-opal[6] to obtain evaluation metrics, which included completeness, purity, and the F1 score.

The calculation methodologies for precision, recall, and the F1 score are based on true positives (TP), false positives (FP), and false negatives (FN), as follows:

Completeness = TP/(TP+FP), defined as the ratio of taxa correctly predicted to be present to all taxa present at the selected taxonomic rank.

Purity = TP/(TP+FN), defined as the ratio of taxa correctly predicted to be present to all predicted taxa at the selected taxonomic rank.

F1 score = 2×Completeness×Purity / (Completeness+Purity), defined as the harmonic average of completeness and purity at the selected taxonomic rank.

**Runtime Analysis**

This study conducted speed tests in a manner that closely simulates real research conditions. Using files each containing 10,000 V4 region sequences extracted from the Greengenes2 database, we tested the classification speed of various annotation tools across 10 to 100 files. For each tool, we conducted tests using their default annotation parameters on 64 threads (except for setting the maxaccept parameter to 1 for BLAST). All tests were performed on the same platform (Intel(R) Xeon(R) Gold 6230 CPU with 512GB memory).

**Habitat statistics and selection based on big-data**

We collected 275,793 samples (**Table S3**) from the MSE database as prior data to identify common DNA sequences within each habitat. Since the data in MSE are annotated only with Greengenes results, we extracted sequences from the V4, V3V4, and V3V5 regions of both Greengenes and Greengenes2 using 515F/806R[1, 2], 341F/806R[7], and 341F/926R[8] primers. Subsequently, we employed CD-HIT to cluster sequences from these regions in both databases with a 100% similarity threshold, creating a mapping collection between the two. For each collection, we annotated the Greengenes2 sequences and calculated their expected recall rate (**Table S4**). For each habitat, we employed *equation S3-S5* to compute the expected Completeness rate $C$, Purity $\mathrm{rate} P$, F1 score $F$ for species-level annotations using the HWL method:

| $C=\frac{\sum_{i=1}^{n} \frac{M_{i}}{T_{i}}}{n}$ | *(S3)* |
| --- | --- |
| $P= C*C$ | *(S4)* |
| $F=\frac{2*(C*P)}{C+P}$ | *(S5)* |

Here, $n$ is the total number of occurrences of all sequences in all samples of the habitat, $i$ is a certain sequence in the habitat, $M_{i}$ is the annotation with the highest weight in the mapped set for that sequence, and $T_{i}$ is the total number of annotations in the set. This formula allows for a systematic estimation of the average completeness of all sequences in a habitat and the calculation of the F1 score accordingly. Since false-positive data is generally more diverse, resulting in purity typically lower than completeness, purity was set to $R*R$ to evaluate the impact of false-positives on purity consistently.

For sequence mapping sets with annotation conflicts, the LCA method does not output the annotation for that sequence, thus only computing those sequences with unanimous annotations. Due to this characteristic, the theoretical accuracy of LCA is 1; hence the F1 score result for LCA is calculated with *equation S6-S7*:

| $C=\frac{\sum_{i=1}^{n} \left[ \frac{M_{i}}{T_{i}}=1 \right]\cdot\frac{M_{i}}{T_{i}}}{n}$ | *(S6)* |
| --- | --- |
| $F=\frac{2*\left( C*1 \right)}{C+1}$ | *(S7)* |

**Supplementary References**

1. Apprill, A., et al., *Minor revision to V4 region SSU rRNA 806R gene primer greatly increases detection of SAR11 bacterioplankton.* Aquatic Microbial Ecology, 2015. **75**(2): p. 129-137.

2. Parada, A.E., D.M. Needham, and J.A. Fuhrman, *Every base matters: assessing small subunit rRNA primers for marine microbiomes with mock communities, time series and global field samples.* Environmental Microbiology, 2016. **18**(5): p. 1403-1414.

3. Martin, M., *Cutadapt removes adapter sequences from high-throughput sequencing reads.* 2011, 2011. **17**(1): p. 3 %J EMBnet.journal.

4. Callahan, B.J., et al., *DADA2: High-resolution sample inference from Illumina amplicon data.* Nature Methods, 2016. **13**(7): p. 581-+.

5. Magoc, T. and S.L. Salzberg, *FLASH: fast length adjustment of short reads to improve genome assemblies.* Bioinformatics, 2011. **27**(21): p. 2957-2963.

6. Meyer, F., et al., *Critical Assessment of Metagenome Interpretation: the second round of challenges.* Nature Methods, 2022. **19**(4): p. 429-+.

7. Parulekar, N.N., et al., *Characterization of bacterial community associated with phytoplankton bloom in a eutrophic lake in South Norway using 16S rRNA gene amplicon sequence analysis.* Plos One, 2017. **12**(3): p. 22.

8. Ceuppens, S., et al., *Microbial community profiling of fresh basil and pitfalls in taxonomic assignment of enterobacterial pathogenic species based upon 16S rRNA.* International Journal of Food Microbiology, 2017. **257**: p. 148-156.

**Supplementary Figures**

**
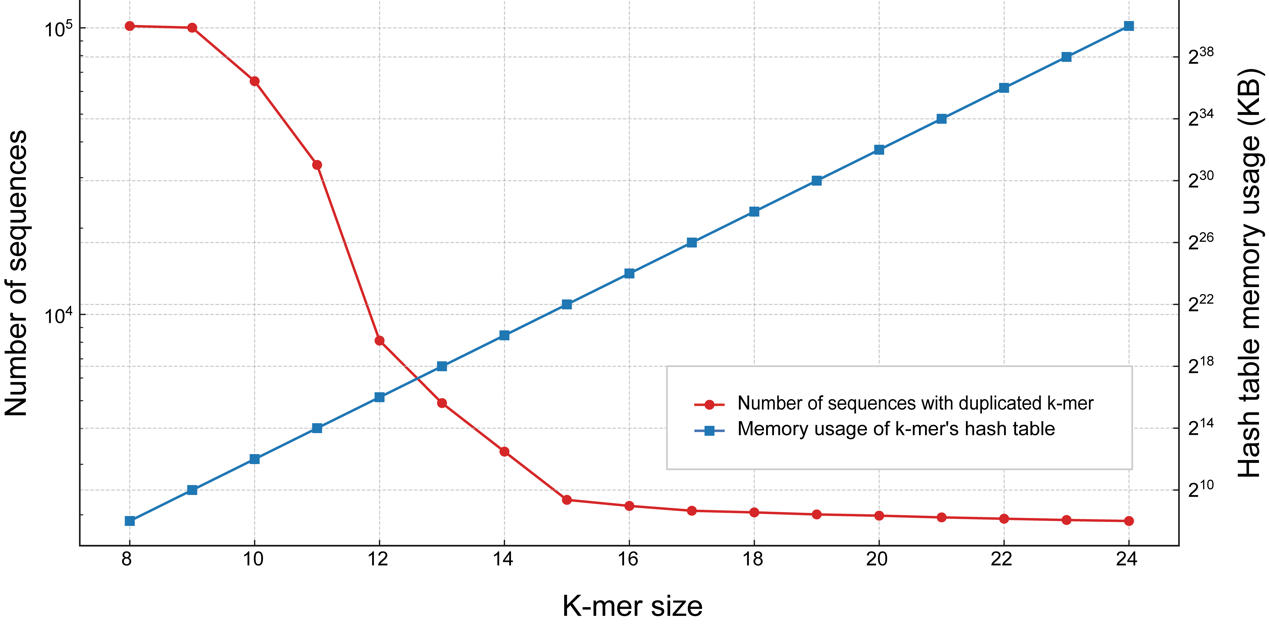
**

**Fig. S1.** **The effect of k-mer size on the number of sequences with duplicated k-mers and the memory usage of the hash table.**


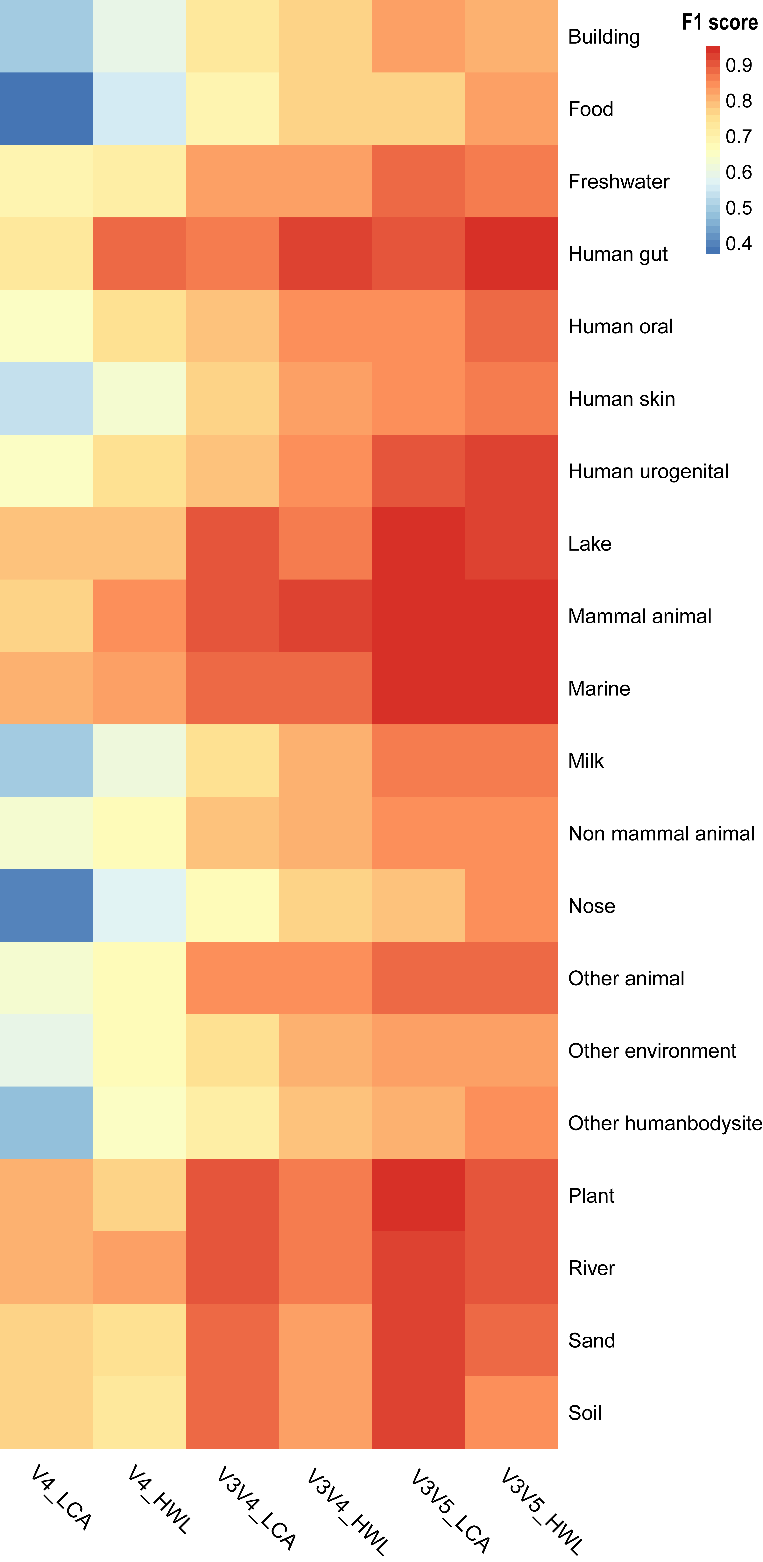


**Fig. S2.** **Heatmap of predicted F1 scores for HWL and LCA across variable regions in different habitats.**


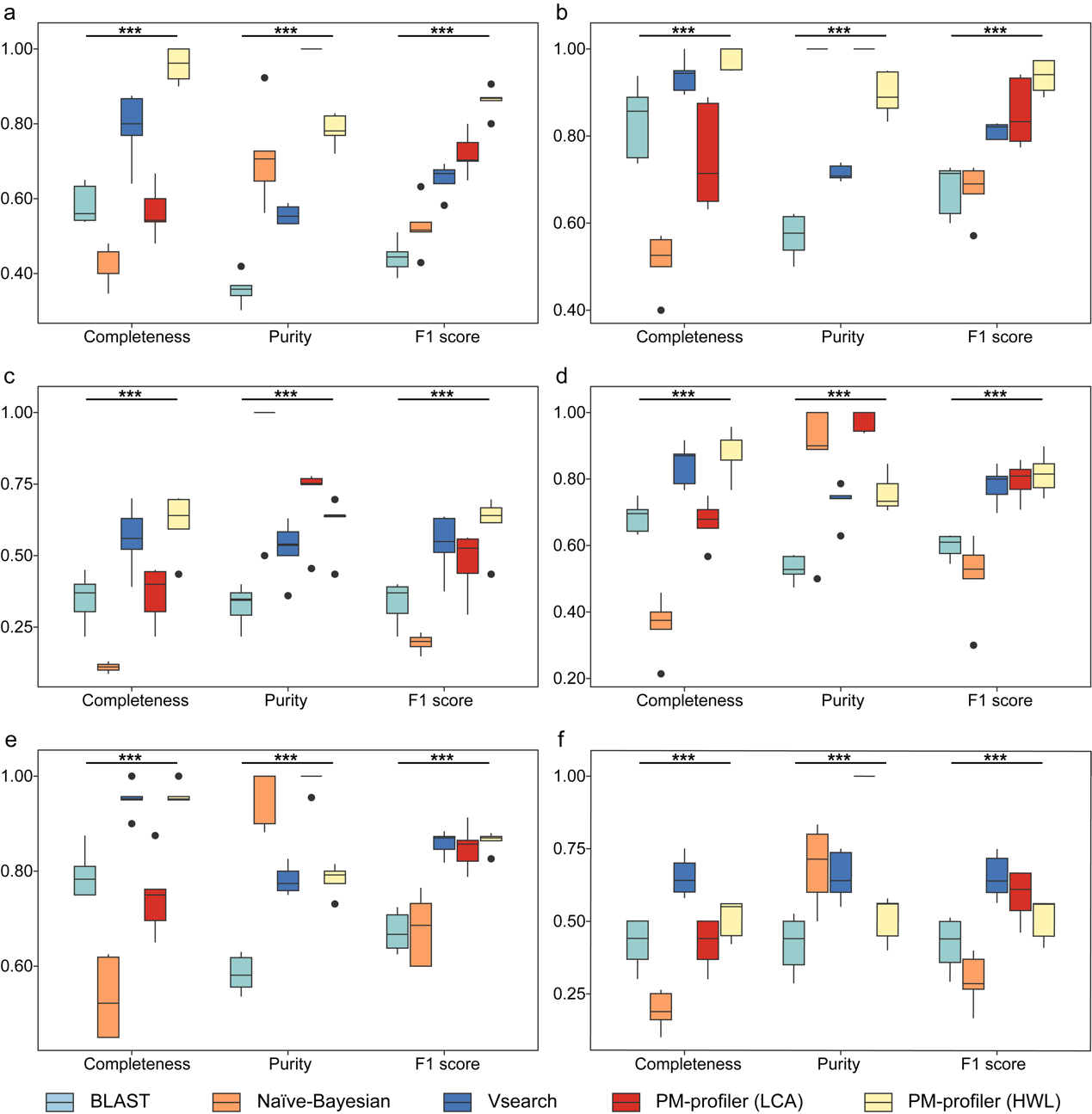


**Fig. S3.** **Species-level taxonomy of different habitats on simulated datasets.** (a) Human Gut. (b) Human Oral. (c) Human Skin. (d) Marine. (e) Mouse Gut. (f) Soil. *** denotes *p-value* < 0.01 by two-tailed rank sum test.

**Supplementary Tables**

**Table S1**. Number of samples used in each habitat.

**Table S2**. Predicted F1 scores for each habitat.

**Table S3**. Species pools of simulated data for each habitat.

**Table S4**. Mock community samples used in the test.
